# Sex-Specific Determinants of the Ketone Body β-Hydroxybutyrate in the General Population

**DOI:** 10.1210/clinem/dgaf587

**Published:** 2025-10-29

**Authors:** Martine G E Knol, Amarens van der Vaart, Lyanne Kieneker, Margery A Connelly, Stephan J L Bakker, Roman-Ulrich Müller, Markus M Rinschen, Ron T Gansevoort, Maatje D A van Gastel

**Affiliations:** Department of Internal Medicine, Division of Nephrology, University of Groningen, University Medical Center Groningen, 9713 GT Groningen, the Netherlands; Department of Internal Medicine, Division of Nephrology, University of Groningen, University Medical Center Groningen, 9713 GT Groningen, the Netherlands; Department of Internal Medicine, Division of Nephrology, University of Groningen, University Medical Center Groningen, 9713 GT Groningen, the Netherlands; Labcorp, Morrisville, NC 27560, USA; Department of Internal Medicine, Division of Nephrology, University of Groningen, University Medical Center Groningen, 9713 GT Groningen, the Netherlands; Department II of Internal Medicine and Center for Molecular Medicine Cologne, Faculty of Medicine and University Hospital Cologne, University of Cologne, 50937 Cologne, Germany; Department of Biomedicine, Aarhus University, 8000C Aarhus, Denmark; III Department of Medicine and Hamburg Center for Kidney Health, University Medical Center Hamburg-Eppendorf, 20251 Hamburg, Germany; Department of Internal Medicine, Division of Nephrology, University of Groningen, University Medical Center Groningen, 9713 GT Groningen, the Netherlands; Department of Internal Medicine, Division of Nephrology, University of Groningen, University Medical Center Groningen, 9713 GT Groningen, the Netherlands

**Keywords:** β-hydroxybutyrate, ketone bodies, sex differences, general population

## Abstract

**Context:**

Being in a state of ketosis has been associated with numerous positive health outcomes and is currently studied in various medical fields. However, the influence of factors other than diet on ketone body levels in the general population has not yet been investigated.

**Objective:**

This study aimed to investigate factors that are associated with plasma levels of the ketone body β-hydroxybutyrate (BHB) levels in a general population cohort.

**Methods:**

We included 6102 participants with available plasma BHB data from the Prevention of Renal and Vascular End-stage Disease (PREVEND) population-based cohort study. Fasting plasma BHB was measured using nuclear magnetic resonance spectroscopy. Determinants of BHB concentrations were identified using univariable and stepwise backward regression analyses.

**Results:**

Women had significantly higher BHB levels than men (123 [interquartile range (IQR) 94-175] vs 119 [IQR 92-164] µmol/L, respectively; *P* < .001). In women, hormonal status was the most significant determinant of BHB levels. In a multivariable-adjusted model, oral contraceptive (OC) use was associated with a 45% increase in BHB concentration, whereas postmenopausal status was associated with an 11% decrease compared to premenopausal women without OC use. In men, age was the most significant determinant, with a 1% increase in BHB levels per year. In both sexes, other determinants of higher BHB levels were lower protein intake, habitual alcohol use, higher N-terminal pro-B-type natriuretic peptide, and a higher free thyroxine level.

**Conclusion:**

A complex interplay of metabolic, hormonal, and lifestyle factors are associated with BHB levels in the general population, with distinct sex differences. In women, hormonal status was the most important factor, while in men increasing age was the most significant factor affecting BHB levels.

Being in a state of ketosis has been associated with numerous positive health outcomes, such as improved metabolic health (1, 2). Ketone bodies are an alternative energy source in a low-carbohydrate state, such as prolonged fasting, post exercise, or starvation (1). Ketone bodies are formed in the liver, where production is stimulated by glucagon and inhibited by insulin (1). They consist of acetoacetate (AcAc), which is rapidly converted to β-hydroxybutyrate (BHB) and decarboxylated to acetone. Beyond their role in providing cells with energy, ketone bodies—particularly BHB—function as signaling molecules through binding to several G protein–coupled receptors. BHB can suppress oxidative stress, inhibit inflammation, and reduce fibrosis (1, 3, 4). Ketone bodies can be measured via nuclear magnetic resonance (NMR) spectroscopy, a validated and reliable method (5).

A ketogenic state is currently being studied as a metabolic treatment option for a variety of diseases. In patients with obesity and type 2 diabetes (T2D), a ketogenic diet improved metabolic parameters, including weight, lipid, and glucose levels (2). Furthermore, the effects of ketone bodies are studied in cardiology, nephrology, neurology, and oncology. For example, a ketogenic diet has been implicated to improve tolerance against cardiac ischemia (6), to reduce disease severity of hypertensive and diabetic kidney disease (7-9), to enhance cognitive function of patients with Alzheimer disease (10), and to inhibit tumor growth in several preclinical models (11). In addition, recently, two trials studying the effect of a ketogenic diet were performed in patients with autosomal dominant polycystic kidney disease, and these interventions were deemed feasible and safe (12, 13).

Understanding determinants of ketone body levels is crucial for explaining the variability in ketone body levels observed among participants in dietary intervention trials and may also provide ways to increase the potency of ketogenic dietary protocols. Besides diet, factors such as sex, body mass index (BMI), and T2D may influence ketone body levels (2, 5, 14). However, these factors have not been studied in conjunction. It is therefore still unclear to which extent these factors influence ketone body levels. Our primary objective was to identify demographic, lifestyle, and biological factors associated with BHB concentrations in a cohort of predominantly healthy individuals. Our second objective was to evaluate specific factors associated with BHB concentrations separately in men and women. Determining unique predictors of BHB concentrations may help to identify which interventions or preventive strategies are most promising to be used when targeting ketone metabolism. Since some predictors differ between men and women, considering sex-specific differences may be important when tailoring such approaches in relevant patient groups.

## Materials and Methods

### Study Cohort

The present study used data from the Prevention of Renal and Vascular End-stage Disease (PREVEND), a prospective population-based cohort study. The design of the cohort has been described elsewhere (15). In summary, from 1997 to 1998, all inhabitants of Groningen, the Netherlands, between ages 28 and 75 years were invited to participate. Inhabitants who were pregnant or had insulin-dependent diabetes were excluded from participation. In total, 8592 participants completed the screening program. The PREVEND study was approved by the medical ethics committee of the University Medical Center Groningen and was performed in accordance with the Declaration of Helsinki guidelines. Written informed consent was obtained from all participants.

For the present analyses, data from participants who completed the second screening round (N = 6894) between 2001 and 2003 were used, due to the availability of NMR blood sample analyses for these participants. We excluded participants with unknown BHB concentration (N = 360), extreme outliers of BHB (defined as a value 3 times the SD higher and lower than the average value, N = 25), and participants who were nonfasting or had an insulin concentration greater than 25 mIU/L, as indicators of a nonfasting state (N = 407), leaving 6102 participants for the analyses. For the linear regression analyses, participants were removed listwise (N = 1154), leaving 4948 participants for these analyses (Supplementary Fig. S1 (16)).

### Laboratory Measurements

Fasting blood samples were taken from the participants in the morning after a self-reported overnight fast. EDTA-anticoagulated plasma samples were prepared by centrifugation at 4 °C and were stored at −80 °C until analysis. Triglycerides, total cholesterol, and high-density lipoprotein (HDL) cholesterol were measured as previously described (17). Fasting plasma glucose was measured by dry chemistry (Eastman Kodak). Fasting insulin was measured with an AxSYM autoanalyzer (Abbott Diagnostics). Plasma C-peptide levels were determined using an electrochemiluminescent immunoassay on a Cobas e602 analyzer (Roche Modular E, Roche Diagnostics). Free thyroxine (FT4) was measured using the Roche Modular E170 Analyzer electrochemiluminescent immunoassays (Roche Diagnostics). Plasma N-terminal pro-B-type natriuretic peptide (NT-proBNP) levels were measured using the Elecsys 2010 analyzer (Elecsys proBNP, Roche Diagnostics). Growth hormone (GH) was measured with a high-sensitivity chemiluminescence sandwich immunoassay similar to one previously described (SphingoTec GmbH) (18). Serum creatinine was determined by Kodak Ektachem dry chemistry (Eastman Kodak), and serum cystatin C level by nephelometry (BN II N) (Dade Behring Diagnostic). Estimated glomerular filtration rate (eGFR) was calculated using the 2012 combined creatinine-cystatin C–based Chronic Kidney Disease Epidemiology Collaboration equation (19).

Participants provided two consecutive 24-hour urine samples following both oral and written instructions. Sodium and urea concentrations were measured in these samples. To calculate excretion per 24 hours, the concentrations of sodium and urea were multiplied by the total urine volume (in liters) collected over 24 hours. For the analyses, the average of the results from the two 24-hour urine collections was used. Subsequently, protein intake was calculated using the Maroni equation (20) = [urea excretion (mmol/24 h) × 0.028 + 0.031 × body weight (kg)] × 6.25.

### Ketone Body Quantification

Plasma BHB, AcAc, and acetone were measured using a Vantera Clinical Analyzer (Labcorp), a fully automated, high-throughput, 400-MHz proton (1H) NMR spectroscopy platform. NMR measures BHB, AcAc, and acetone with a good clinical performance with a mean interassay coefficient of variation percentage of 5.8, 6.9, and 7.0, and a mean intra-assay coefficient of variation percentage of 5.2, 5.1, and 5.0, at low, medium, and high concentrations, respectively (5). The long-term stability of BHB in samples stored frozen for more than 6 years was good (<1.5% bias). We chose BHB as the primary outcome measure due to the limited long-term stability of AcAc and acetone (−13.8 and 19.1% bias, respectively), because BHB is the most abundant ketone body during fasting, and because it is the most extensively studied ketone body given its role as signaling metabolite (1). More details about the ketone body measurement are reported elsewhere (5). Compared to other methods, such as the semi-quantitative nitroprusside test, NMR offers more reliable and accurate measurements of ketone bodies, particularly at low concentrations (5, 21).

### Clinical Measurements

All participants completed a questionnaire about cardiovascular and kidney disease history, current smoking status, alcohol use, exercise frequency, medical history, and medication use. Women also reported their menopausal status and the use of oral contraception (OC). Alcohol use was categorized in the following categories: abstention (no alcohol consumption), 1 to 4 units/month, 2 to 7 units/week, 1 to 3 units/day, or >3 units/day. Subsequently, we grouped the categories into never drinking, occasional drinking (1-4 units/month and 2-7 units/week), and habitual drinking (1-3 units/day and >3 units/day). Blood pressure was measured as the mean of the last 2 recordings using an automatic Dinamap XL Model 9300 series device (Johnson-Johnson Medical). BMI was calculated by dividing the weight in kilograms by the height in square meters. Waist circumference was measured directly on the skin at the natural indentation located between the tenth rib and the iliac crest. In cases where no indentation was present, the measurement was taken at the middle between the navel and the rib cage.

T2D was defined as fasting glucose level of 7.0 mmol/L or greater, a nonfasting glucose level of 11.1 mmol/L or greater, or the use of antidiabetic medication either self-reported or from pharmacy data. Hypertension was described as a systolic blood pressure of 140 mm Hg or greater, diastolic blood pressure of 90 mm Hg or greater, or the use of antihypertensive medication. Hyperthyroidism was defined as a thyrotropin (TSH) level less than 0.5 mU/L and FT4 greater than 19.5 pmol/L and hypothyroidism as TSH greater than 4.0 mU/L and FT4 less than 11.0 pmol/L according to the University Medical Center Groningen guidelines. A cutoff of NT-proBNP greater than 500 ng/L was selected to indicate heart failure, as levels above this threshold are generally associated with a high likelihood of heart failure across all age groups (22).

### Statistical Analysis

We used R version 4.0.5 for the analyses. A 2-sided *P* value with an α of .05 was considered statistically significant. Normally distributed data are presented as means ± SD, skewed data as medians and interquartile range (IQR), and categorical data as frequencies with percentages. All continuous independent and dependent variables that were not normally distributed were logarithmically transformed to meet the assumptions for linear regression analysis.

Baseline characteristics were stratified by sex-adjusted quartiles of BHB, as BHB levels were significantly higher in women than in men, consistent with the previous literature (23). Differences across quartiles were assessed using one-way analysis of variance for normally distributed variables, the Kruskal-Wallis test for nonnormally distributed variables, and the chi-square test for categorical variables. Trends across quartiles were evaluated using a linear polynomial contrast in one-way analysis of variance for normally distributed variables, the Jonckheere-Terpstra test for nonnormally distributed variables, and the linear-by-linear association test for categorical variables.

For the primary objective, possible determinants of BHB concentration were explored using univariable linear regression and multivariable backward stepwise regression analyses. Based on the literature, we took the following determinants into account: sex, menopause status, OC use, age, BMI, glucose, C-peptide, T2D, eGFR, sodium excretion as a proxy for sodium intake, protein intake, smoking, alcohol, NT-proBNP, β-blocker use, FT4, GH, and exercise status (1, 2, 5, 9, 14, 24-34). In an integrated manner, using a stepwise backward multivariable linear regression analysis, the associations between the variables of interest and BHB as dependent variable were tested. The previously mentioned variables were added to the model as independent variables in case the *P* value was less than .10 in the univariable linear regression analyses. All stepwise backward analyses were adjusted for age and sex. *P* greater than .05 was used to exclude the variables stepwise from the models. Before exclusion, continuous variables were tested for nonlinear associations by adding the squared variable to the model. The variables in the final model were tested for nonlinear associations and interactions with sex. Since we found a strong interaction between age and sex for their association with BHB, we performed further analyses stratified by sex as a secondary objective. Exponential coefficients and 95% CIs are shown since the dependent variable BHB is log_2_ transformed. Participants were removed listwise in case of missing data (N = 1154), leaving 4948 participants for these analyses (see Supplementary Fig. S1 (16)).

## Results

### Participant Characteristics

In total, 6102 participants were analyzed with a mean age of 53.6 ± 12.1 years with a median BHB of 122 µmol/L (IQR 93-169 µmol/L); 3098 (50.8%) were women. In women, the median BHB concentration was 123 µmol/L (IQR 94-175 µmol/L), significantly higher than in men (119 µmol/L, IQR 92-164 µmol/L; *P* < .001), although the absolute difference was relatively small. Participant characteristics are given in sex-specific quartiles of BHB, and most characteristics were significantly different across these quartiles (Table 1). The following characteristics increased significantly per increasing quartile of BHB: age, hypertension, SBP, NT-proBNP, T2D, FT4, and OC use. Conversely, eGFR and sodium intake declined per quartile of BHB.

**Table 1.** Participants characteristics of the overall study cohort as well as according to sex-adjusted quartiles of plasma β-hydroxybutyrate

		Quartiles of baseline BHB, µmol/L					
	Total	F: < 93.5 M: < 92.4	F: 93.5-123.0 M: 92.4-119.0	F: 123.1-175.0 M: 119.1-164.0	F: > 175.0 M: > 164.0	*P*	*P* for trend
Total, n	6102	1526	1525	1525	1526		
BHB, µmol/L	122 (93-169)	77 (67-86)	106 (99-114)	140 (130-154)	239 (195-320)	<.001	<.001
F, n (%)	3098 (50.8%)	775 (50.8%)	774 (50.8%)	774 (50.8%)	775 (50.8%)	≥.999	≥.999
Age, y	53.6 ± 12.1	49.9 ± 11	53.3 ± 11.6	55.4 ± 11.9	55.9 ± 12.7	<.001	<.001
Weight, kg	79.1 ± 14.1	77.2 ± 13.3	80.2 ± 13.9	80.4 ± 14.4	78.5 ± 14.4	<.001	.01
BMI	26.5 ± 4.2	25.5 ± 3.6	26.8 ± 4.2	27 ± 4.3	26.6 ± 4.4	<.001	<.001
eGFR, mL/min/1.73 m^2^	92.2 ± 17.1	96.8 ± 15.3	92.7 ± 16.6	90.5 ± 17.2	88.9 ± 18.1	<.001	<.001
Hypertension, n (%)	1932 (31.7%)	329 (21.6%)	465 (30.5%)	550 (36.0%)	588 (38.5%)	<.001	<.001
SBP, mm Hg	125.8 ± 18.7	121.5 ± 16.5	125.3 ± 17.7	127.6 ± 19.1	128.9 ± 20.3	<.001	<.001
NT-proBNP, ng/L	42 (22-81)	36 (19-68)	39 (20-75)	43 (22-84)	52 (27-103)	<.001	<.001
Type 2 diabetes, n (%)	330 (5.4%)	22 (1.4%)	61 (4.0%)	102 (6.7%)	145 (9.5%)	<.001	<.001
Glucose, mmol/L	4.8 (4.4-5.3)	4.7 (4.4-5.1)	4.8 (4.4-5.3)	4.8 (4.5-5.4)	4.8 (4.4-5.4)	<.001	<.001
Insulin, mU/L	8.0 (5.7-11.7)	6.9 (5.1-9.6)	8.3 (6-11.9)	8.8 (6.1-12.9)	8.2 (5.7-12.1)	<.001	<.001
C-peptide, pmol/L	728 (568-944)	657 (537-827)	742 (584-951)	780 (601-1029)	745 (562-979)	<.001	<.001
Total cholesterol, mmol/L	5.4 ± 1.0	5.3 ± 1.0	5.5 ± 1.1	5.5 ± 1.0	5.4 ± 1.0	<.001	.03
HDL cholesterol, mmol/L	1.3 ± 0.3	1.3 ± 0.3	1.2 ± 0.3	1.2 ± 0.3	1.3 ± 0.3	<.001	.001
Triglycerides, mmol/L	1.1 (0.8-1.6)	1.0 (0.7-1.3)	1.1 (0.8-1.6)	1.2 (0.9-1.7)	1.1 (0.8-1.6)	<.001	<.001
FT4, pmol/L	15.6 ± 2.4	15.5 ± 2.2	15.6 ± 2.3	15.6 ± 2.4	16.0 ± 2.5	<.001	<.001
Growth hormone, ng/mL	0.28 (0.06-1.14)	0.26 (0.06-1.29)	0.24 (0.06-1.17)	0.27 (0.06-1.07)	0.34 (0.09-1.1)	.009	.03
Sodium intake, mmol/24 h	144 ± 54.9	147.9 ± 54.5	145.7 ± 55	144.2 ± 54.4	138.1 ± 55.3	<.001	<.001
Protein intake, g/24 h	80 ± 21.1	81.4 ± 21.2	81.6 ± 21.3	80.3 ± 20.5	76.7 ± 21.2	<.001	<.001
Alcohol consumption, n (%)						<.001	.0001
Never	1496 (24.5%)	377 (24.7%)	391 (25.6%)	376 (24.7%)	352 (23.1%)		
Occasional	2942 (48.2%)	766 (50.2%)	762 (50.0%)	738 (48.4%)	676 (44.3%)		
Habitual, ≥1× daily	1606 (26.3%)	365 (23.9%)	361 (23.7%)	398 (26.1%)	482 (31.6%)		
Missing	58 (1.0%)	18 (1.2%)	11 (0.7%)	13 (0.9%)	16 (1.1%)		
Smoking current, yes n (%)	1689 (27.7%)	423 (27.7%)	415 (27.2%)	433 (28.4%)	418 (27.4%)	.9	.9
Sport, n (%)						<.001	.003
No exercise	1284 (21%)	289 (18.9%)	344 (22.6%)	344 (22.6%)	307 (20.1%)		
≤1× per wk	3733 (61.2%)	903 (59.2%)	908 (59.5%)	963 (63.1%)	959 (62.8%)		
≥ 2× per wk	1008 (16.5%)	311 (20.4%)	256 (16.8%)	199 (13.0%)	242 (15.9%)		
Missing	77 (1.26%)	23 (1.51%)	17 (1.11%)	19 (1.25%)	18 (1.18%)		
OC use, yes n (%)	400 (6.6%)	39 (2.6%)	71 (4.7%)	130 (8.5%)	160 (10.5%)	<.001	<.001
Menopause status, n (%)						<.001	.83
Premenopausal	1451 (23.8%)	399 (26.1%)	316 (20.7%)	337 (22.1%)	399 (26.1%)		
Postmenopausal	1582 (25.9%)	355 (23.3%)	444 (29.1%)	422 (27.7%)	361 (23.7%)		
Missing	65 (1.1%)	21 (1.4%)	14 (0.9%)	15 (1.0%)	15 (1.0%)		

Normally distributed data are presented as mean ± SD, nonnormally distributed as median (interquartile range), and categorical variables are presented as frequencies and percentages. *P* values for differences between groups and *P* for trend were tested with, respectively, one-way analysis of variance including testing for a linear polynomial contrast for normally distributed data; Kruskal-Wallis and Jonckheere-Terpstra test for nonnormally distributed data; and Pearson chi-square and linear-by-linear association test for categorical data. Protein intake was calculated in grams per 24 hours, using the Maroni formula, from urea excretion in 24-hour urine collections (20).

Abbreviations: BHB, β-hydroxybutyrate; BMI, body mass index; eGFR, estimated glomerular filtration rate; F, female; FT4, free thyroxine; HDL, high-density lipoprotein; M, male; NT-proBNP, N-terminal pro-B-type natriuretic peptide; OC, oral contraceptive; SBP, systolic blood pressure.

### Linear Regression Analyses

#### Primary objective

In the overall cohort, the following variables were significantly associated with BHB in the univariable analyses and were included in the stepwise backward model: sex, age, BMI, glucose, C-peptide, T2D, eGFR, sodium intake, protein intake, daily alcohol use, NT-proBNP, β-blockers, FT4, and GH. Results can be found in Supplementary Table S1 (16). In the stepwise backward model, an interaction effect was found between sex and age (B = 0.899 [95% CI, 0.873-0.925]; *P* < .001) for their association with BHB. Increasing age was associated with a higher BHB level in men compared to women. Given these significant interactions with sex, we conducted separate analyses for women and men.

#### Secondary objective: associations of β-hydroxybutyrate concentration in men

**Figure 1.**
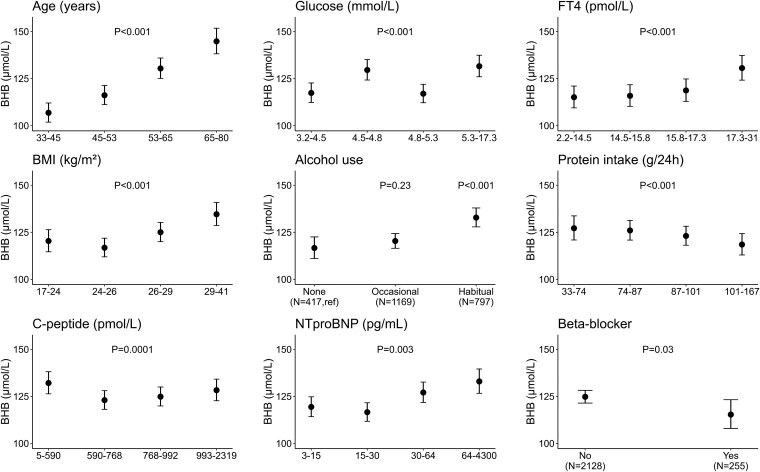
Significant determinants of the plasma β-hydroxybutyrate (BHB) concentrations in men. The BHB concentrations were estimated per quartile of the significant continuous or categorical distributed variables. The estimates were determined using the final stepwise backward model including all variables and including the quartiles of each specific variable. Subsequently, the estimates were back-log transformed to show the effect in BHB concentration in µmol/L. The error bars show the 95% CI. The *P* values of the stepwise backward model are included in the figure. For alcohol use, the reference group is no alcohol.

In men, univariable linear regression analyses identified several significant determinants of BHB concentration: age, BMI, glucose, C-peptide, T2D, eGFR, sodium intake, protein intake, habitual alcohol use, NT-proBNP, β-blockers, FT4, GH (all *P* < .001), and exercise 2 or more times per week (*P* = .03). These variables were subsequently included in a stepwise backward regression analysis. The results can be found in Table 2 and Fig. 1. Fig. 1 ranks these determinants from the most to the least significant contributors to the model. The final multivariable model explained 13% of the variation in BHB concentration among men. The results showed that age was the most significant contributor to the model, with an increase of 12.4 years in age being associated with 12% higher BHB concentrations (B = 1.124 [95% CI, 1.097-1.152]; *P* < .001). Habitual alcohol use was associated with an increase of 14% in BHB levels (B = 1.140 [95% CI, 1.077-1.206]; *P* < .001). Additionally, higher levels of BMI, glucose, NT-proBNP, and FT4 were associated with increased BHB concentrations. In contrast, β-blocker use was associated with a decrease of 7% in BHB levels (B = 0.926 [95% CI, 0.866-0.991]; *P* = .03). Furthermore, higher protein intake was associated with lower BHB concentrations.

**Table 2.** Univariable regression analyses and stepwise backward analyses of determinants of the dependent variable β-hydroxybutyrate in men and women

	Men (N = 2383)				Women (N = 2565)			
	Univariable analyses		Stepwise backward analysis		Univariable analyses		Stepwise backward analysis	
	B* (95% CI)	*P*	B* (95% CI)	*P*	B* (95% CI)	*P*	B* (95% CI)	*P*
Hormonal status (ref: premenop.)								
Premenopausal OC use	—		—		1.412 (1.325-1.504)	<.001	1.448 (1.359-1.543)	<.001
Postmenopausal	—		—		1.076 (1.031-1.122)	.0007	0.893 (0.840-0.950)	.0003
Postmenopausal OC use	—		—		1.198 (0.966-1.486)	.10	1.098 (0.891-1.355)	.38
Age, y	1.160 (1.137-1.182)	<.001	1.124 (1.097-1.152)	<.001	1.035 (1.015-1.056)	.0006	1.055 (1.019-1.092)	.002
BMI	1.052 (1.031-1.074)	<.001	1.060 (1.034-1.086)	<.001	1.028 (1.008-1.048)	.006		
Glucose, mmol/L	1.078 (1.056-1.100)	<.001	1.053 (1.032-1.075)	<.001	1.039 (1.018-1.060)	.0003		
C-peptide, pmol/L	1.065 (1.044-1.087)	<.001	0.839 (0.766-0.918)	.0001	1.038 (1.018-1.059)	.0002		
C-peptide^2^			1.026 (1.013-1.040)	.0001				
T2D, yes	1.320 (1.209-1.441)	<.001			1.287 (1.162-1.427)	<.001	1.229 (1.112-1.358)	<.001
eGFR, mL/min/1.73 m^2^	0.890 (0.873-0.908)	<.001			0.953 (0.934-0.971)	<.001	0.965 (0.942-0.990)	.006
Sodium intake, mmol/24 h	0.954 (0.935-0.973)	<.001			0.968 (0.949-0.987)	.001	0.878 (0.808-0.955)	.002
Sodium intake^2^							1.019 (1.007-1.032)	.003
Protein intake, g/24 h	0.945 (0.926-0.965)	<.001	0.953 (0.934-0.973)	<.001	0.956 (0.938-0.975)	<.001	0.971 (0.947-0.996)	.02
Smoking, yes	0.983 (0.939-1.028)	.45			0.982 (0.939-1.026)	.41		
Alcohol use (ref: no alcohol use)								
Occasional	0.985 (0.931-1.043)	.61	1.033 (0.979-1.090)	.23	0.974 (0.931-1.018)	.24	1.003 (0.960-1.047)	.9
Habitual (≥1× daily)	1.108 (1.043-1.176)	.0008	1.140 (1.077-1.206)	<.001	1.062 (1.004-1.124)	.04	1.102 (1.044-1.163)	.0004
NT-proBNP log_2_	1.060 (1.049-1.072)	<.001	1.020 (1.007-1.033)	.003	1.039 (1.023-1.055)	<.001	1.016 (1.000-1.032)	.046
β-blockers	1.115 (1.044-1.190)	.001	0.926 (0.866-0.991)	.03	1.023 (0.954-1.098)	.52		
FT4, pmol/L	1.037 (1.016-1.058)	.0005	1.043 (1.023-1.064)	<.001	1.050 (1.030-1.071)	<.001	1.040 (1.021-1.060)	<.001
GH log_2_	1.029 (1.019-1.039)	<.001			0.981 (0.972-0.990)	<.001	0.957 (0.947-0.968)	<.001
GH log_2_^2^							0.992 (0.988-0.996)	<.001
Exercise (ref: no exercise)								
≤ 1× per wk	0.998 (0.950-1.049)	.9			1.002 (0.951-1.055)	.9		
≥ 2× per wk	0.932 (0.875-0.994)	.03			0.995 (0.929-1.065)	.9		

B*: all coefficients are log back-transformed for interpretation. For continuous variables, the effect per 1-SD increment is used. Adjusted *R^2^* of stepwise backward analysis is 0.13 in men and 0.11 in women. Protein intake was calculated in grams per 24 hours, using the Maroni formula, from urea excretion in 24-hour urine collections (20).

Abbreviations: B, estimate; BHB, β-hydroxybutyrate; BMI, body mass index; eGFR, estimated glomerular filtration rate; FT4, free thyroxine; GH, growth hormone; NT-proBNP, N-terminal pro-B-type natriuretic peptide; OC, oral contraceptives; ref, reference group; T2D, type 2 diabetes.

#### Secondary objective: associations of β-hydroxybutyrate concentration in women

**Figure 2.**
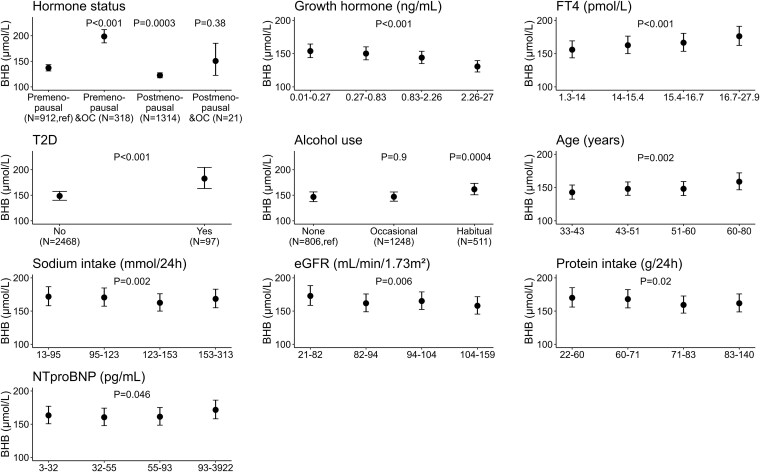
Significant determinants of the plasma β-hydroxybutyrate (BHB) concentrations in women. The BHB concentrations were estimated per quartile of the significant continuous or categorical variables. The estimates were determined using the final stepwise backward model including all variables and including the quartiles of each specific variable. Subsequently, the estimates were back-log transformed to show the effect in BHB concentration in µmol/L. The error bars show the 95% CI. The *P* values of the stepwise backward model are included in the figure. Premenopausal in hormone status, and no alcohol in alcohol use, are the reference groups.

In the univariable linear regression analyses, several variables were significantly associated with BHB concentration in women. These included hormonal status, age, BMI, glucose, C-peptide, T2D, eGFR, sodium intake, protein intake, NT-proBNP, FT4, GH (all *P* < .001), as well as habitual alcohol use (*P* = .04). All of these variables were then included in a stepwise backward regression analysis. The results of the stepwise backward analysis, presented in Table 2 and illustrated in Fig. 2, highlight the determinants that remained significantly associated with BHB concentration. Fig. 2 ranks these determinants from the most to least significant contributors to the model. The final multivariable model explained 11% of the variation in BHB concentration among women. The most significant determinant was OC use in premenopausal women, which was associated with an increase of BHB concentration of 45% compared to premenopausal women not on an OC (B = 1.448 [95% CI, 1.359-1.543]; *P* < .001). Postmenopausal women were associated with a decrease of 11% in BHB concentration compared to premenopausal women not on an OC (B = 0.893 [95% CI, 0.840-0.950]; *P* < .001). Postmenopausal OC use was not significantly associated with BHB. GH was negatively associated with BHB concentration, while an increased FT4 and NT-proBNP were associated with an increased BHB concentration. Habitual alcohol use (>1× unit per day) was significantly associated with an increase of 10% in BHB concentration (B = 1.102 [95% CI, 1.044-1.163]; *P* = .0004), and having T2D was associated with an increase of 23% in BHB (B = 1.229 [95% CI, 1.112-1.358]; *P* < .001). Per 11.6 years, BHB was associated with an increase of 5.5% in BHB levels (B = 1.055 [95% CI, 1.019-1.092]; *P* = .002), which was lower compared to men. Higher sodium intake, protein intake, and eGFR were associated with a lower BHB concentration.

We assessed whether removing women using an OC would influence the association between age and BHB in the stepwise backward model since women using OC have elevated BHB levels in this cohort. In the full cohort of women, the association was B = 1.055 (95% CI, 1.019-1.092; *P* = .002), whereas after excluding women with OC use, the association slightly increased to B = 1.057 (95% CI, 1.019-1.096; *P* = .003) (Supplementary Table S2 and Fig. S2 (16)). To further assess the effect of age, we focused specifically on postmenopausal women, excluding premenopausal women from the analysis. In this subgroup, the association between age and BHB levels became slightly stronger and more significant (B = 1.064 [95% CI, 1.026-1.103]; *P* = .0008).

### Sensitivity Analyses

As a sensitivity analysis, we examined the determinants of total ketone body concentration, AcAc, and acetone using the same approach as for BHB (Supplementary Table S3 (16)). The identified determinants were largely consistent across the different ketone bodies. Sex, age, glucose levels, alcohol consumption, and FT4 were significantly associated with total ketones as well as with each ketone body individually. In addition, there was a significant interaction between sex and age with BHB, total ketone bodies, and AcAc.

In addition, we excluded participants with T2D (N = 231) from the separate analyses for women and men, since having T2D is known to increase BHB levels (5). This exclusion led to only minor changes in the results. In women, glucose was no longer significantly associated with BHB in the univariable analysis, and there were no changes in the stepwise backward analysis. For men, the only difference was that the association between glucose and BHB became nonlinear instead of linear.

As a different measure of obesity, waist circumference was added to the models (35). In men, waist circumference was significantly associated with BHB in the final model (B = 1.057 [95% CI, 1.014-1.102]; *P* = .009), while BMI lost significance (B = 1.017 [95% CI, 0.978-1.058]; *P* = .40). In women, waist circumference was not significantly associated with BHB in the final model.

Participants with insulin levels greater than 25 mIU/L were initially excluded, as this may indicate recent food intake. However, if truly fasting, such levels could also reflect severe insulin resistance. Therefore, a sensitivity analysis including the 271 participants with high insulin who reported to be fasting was performed (Supplementary Table S4 (16)). Results were essentially similar. In men, T2D became significantly associated with BHB, while glucose and C-peptide lost statistical significance.

Kidney function can influence both NT-proBNP and BHB, although the effect on BHB is expected to be minimal, as nearly all BHB is reabsorbed at the low concentrations observed in this cohort, and average kidney function was high. To assess potential confounding, we tested whether including eGFR in the model for men affected the association between NT-proBNP and BHB, given that both NT-proBNP and kidney function were associated with BHB in women. NT-proBNP remained significantly associated with BHB (B: 1.019 [95% CI, 1.005-1.032]; *P* = .006), while eGFR remained not statistically significant, indicating minimal confounding by kidney function.

Furthermore, we repeated the multivariable regression models using TSH instead of FT4 as the primary marker of thyroid function. In women, TSH was negatively associated with BHB, consistent with the inverse relationship between TSH and FT4, as low TSH can reflect increased thyroid hormone activity. Of note, the model including TSH showed a poorer fit based on Akaike information criterion compared to the model with FT4. This supports the use of FT4 instead of TSH, also because FT4 is the effector hormone and thus a more proximal indicator for thyroid hormone activity.

### β-Hydroxybutyrate Levels Across Different Disease States

**Figure 3.**
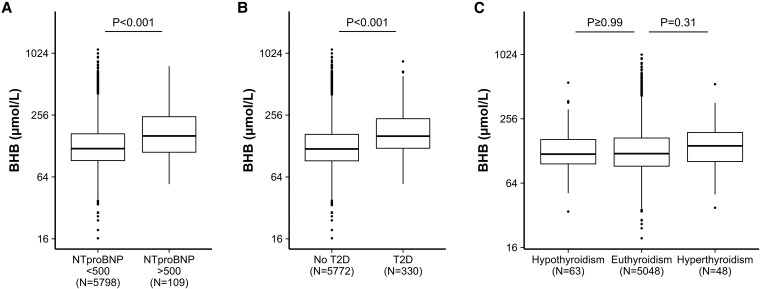
Plasma β-hydroxybutyrate (BHB) levels across different disease categories: A, heart failure; B, type 2 diabetes; and C, thyroid dysfunction. Box plots show BHB concentrations stratified by disease categories. *P* values indicate statistical differences across groups.

We assessed BHB levels across different diseases: possible indication of heart failure (NT-proBNP >500 ng/L), T2D, and hyperthyroidism/hypothyroidism. The results are displayed in Fig. 3. Since the groups were relatively small in the disease groups, both sexes were analyzed together, and all available data were used. There were no statistically significant interactions between sex and the different disease states, nor with age and the different disease states. In total, 109 participants had an elevated NT-proBNP level. The median BHB concentration was 160 µmol/L (IQR 111-247 µmol/L) in the elevated NT-proBNP level group, compared to 121 µmol/L (IQR 92-168 µmol/L) in the not-elevated group (*P* < .001). Participants with T2D (N = 330) had a median BHB level of 160 µmol/L (IQR 122-236 µmol/L) compared to 120 µmol/L (IQR 92-166 µmol/L) in the participants without T2D (*P* < .001). A total of 48 participants had hyperthyroidism based on a low TSH and an elevated FT4 level. In this group, the BHB levels were 143 µmol/L (IQR 102-190 µmol/L) higher although not significantly different compared to euthyroid participants, who had a median BHB concentration of 121 µmol/L (IQR 92-169 µmol/L) (*P* = .30). There was no difference between the participants with suspected hypothyroidism and euthyroid participants (*P* = ≥.999).

## Discussion

In the present study, we investigated possible determinants of BHB concentration in a comprehensive, integrated analysis. To our knowledge, this study is the first to explore the determinants of ketone body concentration in a large general population cohort. Consistent with the previous literature, we found that BHB levels were significantly higher in women than in men (23, 36). Interestingly, we observed an interaction between sex and age on BHB levels. In men, age was more strongly associated with BHB than in women. In men and women, overlapping determinants of BHB levels were protein intake, habitual alcohol use, NT-proBNP, and FT4. In women, the strongest associations with BHB levels were found for hormonal status. OC use in premenopausal women was associated with higher BHB levels and postmenopausal status with lower BHB levels in the multivariable-adjusted model. In men, increasing age played the most substantial role.

At the time of the study visits, the ketogenic diet was not as popular as it is today (37). The relatively low levels of ketone bodies in the participants suggest that these individuals were unlikely to have been following a ketogenic diet. Fasting ketone body concentrations are below 0.5 mmol/L in the general population following a typical Western diet, which is in line with our median BHB concentration of 0.122 mmol/L (1, 24). Nutritional ketosis, for example, during a ketogenic diet, is generally considered to result in BHB levels of 0.5 to 3 mmol/L, whereas BHB levels can rise to 20 mmol/L during diabetic ketoacidosis (12, 24). Therefore, the level of ketone bodies observed in our study was most likely due to other factors than adherence to a ketogenic diet.

Sex differences were evident in the determinants of BHB concentration, with distinct patterns observed between men and women. In men, being older was associated with an increased BHB concentration, likely due to age-related glucose intolerance and insulin insensitivity, both of which can influence ketogenesis (5). In women, the most significant factor associated with BHB concentration was hormonal status, defined by menopausal status and OC use. Women using OCs had higher BHB levels compared to premenopausal women not using OCs. In contrast, postmenopausal women were associated with relatively lower BHB concentrations in the final model. These findings suggest that sex-hormone levels may influence BHB concentrations. An animal study supports this hormonal effect on ketogenesis (38), while in humans, studies examining OC use and ketogenesis have yielded conflicting results. One study showed that during the initial fasting phase, OC use in premenopausal and postmenopausal women could increase BHB levels (39). Conversely, a large Finnish cohort study found no significant differences in ketone body levels with OC use, while free fatty acids (FFAs), the substrate for ketone bodies, were significantly elevated on OC (40). Nevertheless, our findings indicate that female sex hormones, through OC use and menopause status, may play a role in influencing ketogenesis.

An increase in age was associated with higher BHB levels both in women and men, which contrasts with some previous findings in the literature. For example, a study comparing children, women, and men found that children exhibited the highest ketone body levels (41). Moreover, a preclinical study showed that aged mice had reduced ketogenesis (42). However, in both studies longer fasting periods were used (30-86 hours, and 24 hours, respectively), whereas in our study participants had only a mild overnight fast. It is possible that under prolonged fasting conditions, the age-related increase in BHB we observed could be attenuated. We interpret our findings as reflecting age-related decreased insulin sensitivity and impaired glucose tolerance (5).

With respect to associations between disease states and BHB, we observed significant associations with T2D, NT-proBNP, FT4, and kidney function. In women, having T2D was significantly associated with BHB concentration, whereas in men glucose concentration and C-peptide were significantly associated. A possible explanation could be that more men were still undiagnosed compared to women. It is known that patients with T2D tend to have mildly elevated ketone body levels (5), a pattern also observed in our cohort. Glucose levels and ketogenesis are regulated by the same hormones: glucagon and insulin. When glucose levels are low, glucagon increases, stimulating ketogenesis, while reduced insulin levels promote lipolysis and the release of FFAs, which are the primary substrates for ketone bodies (1). However, due to insulin resistance, this metabolic balance can shift. Paradoxically, insulin resistance has been described before as having an inverse relationship with BHB concentrations, while in T2D, BHB levels are mildly elevated (5). This effect might depend on the amount of insulin produced and the degree of insulin resistance, which could explain the associations between glucose and C-peptide and BHB concentration in men.

BMI was positively associated with BHB levels in men. In contrast, previous studies showed that patients and mice with obesity have reduced BHB levels (43, 44). This discrepancy might depend on the degree of obesity since, in the aforementioned study, the average BMI of patients with obesity was 46, much higher than in the cohort under study with an average BMI of 26.6 ± 4.4 in the highest quartile of BHB (44). Notably, a higher waist circumference, representing central adiposity, was more strongly associated with higher BHB levels than BMI in men. This suggests that abdominal fat distribution, rather than overall body mass, may play a more significant role in determining BHB levels. Patients with T2D often have higher BMIs than those without T2D, which could partially explain the positive association between BMI and BHB levels. Thus, the BHB levels might depend on glucose homeostasis and insulin resistance rather than on the degree of obesity itself.

We found a positive association in both sexes between NT-proBNP and BHB. A positive association between NT-proBNP and ketone bodies has been previously reported in the PREVEND cohort (33), as well as in a different cohort (45). It is hypothesized that ketone bodies may serve as an alternative energy source for the failing heart, with natriuretic peptides like NT-proBNP potentially promoting lipolysis, leading to an increase in FFAs and subsequent ketogenesis (46). We additionally establish that NT-proBNP remains significantly associated with BHB in a stepwise backward model. Furthermore, higher FT4 levels were significantly associated with elevated BHB concentrations, with participants suspected of having hyperthyroidism showing increased BHB levels compared to healthy controls and those with hypothyroidism. Elevated BHB levels in hyperthyroid patients have been reported previously in epidemiological studies (47, 48), while experimental studies suggested that FT4 may affect both lipolysis and ketogenesis but is modulated by insulin levels (26). Finally, kidney function was significantly associated with BHB in women. At the low endogenous concentrations present in our cohort, reabsorption is effectively complete and renal clearance negligible (49); thus, kidney function is unlikely to directly influence circulating BHB. The observed association may instead reflect residual confounding by sex-specific differences in lipid metabolism and insulin resistance (23, 50-52). Nevertheless, lower kidney function might lead to higher plasma levels of BHB when BHB levels are increased such as during a ketogenic diet or fasting.

Regarding lifestyle factors, we found statistically significant associations between alcohol use, sodium intake, and protein intake, while exercise showed no association with BHB. Habitual alcohol drinkers (>1 drink daily) had higher ketone levels compared to occasional or nondrinkers. This finding can be attributed to ethanol metabolism in the liver, which alters the NADH/NAD⁺ ratio, impairs gluconeogenesis, enhances ketoacid production, and promotes the conversion of AcAc to BHB, leading to increased BHB levels (53). In addition, alcohol use can lead to alcoholic ketoacidosis, a severe condition linked to chronic alcohol use or binge drinking with minimal food intake (53). Conversely, increased protein intake and sodium intake were associated with reduced BHB levels. Sodium and protein intake correlated with each other, which might explain the negative association between salt intake and BHB. Excessive protein intake may trigger gluconeogenesis, leading to increased glucose levels that could potentially interfere with ketogenesis (54), which could explain the negative association between protein intake and BHB levels. However, in our cohort this association was weak, suggesting that protein intake alone is unlikely to be a major determinant of BHB variation. Carbohydrate intake and meal timing are also contributors to ketone body concentrations; however, detailed dietary records to assess carbohydrate intake and meal timing were not available. Plasma samples were collected after an overnight fast, which standardized meal timing to some extent. Lastly, we did not find a significant effect on regular exercise and BHB concentration. During exercise, especially prolonged and intense, ketone body levels rise and stay elevated several hours post exercise (31). We hypothesize that we did not observe this effect in our study because the blood samples for BHB measurement were taken in the morning after an overnight fast, making it unlikely that people could have exercised in the hours before blood withdrawal to a level meeting the required intensity to affect BHB.

The strengths of the present study are the use of a large and well-phenotyped general population cohort with access to numerous variables possibly associated with BHB, and the reliable method of measuring the ketone bodies. Also, some limitations must be acknowledged. Due to the cross-sectional design of the study, causality cannot be inferred with certainty. In addition, residual confounding can occur. The *R^2^* of the final models were relatively small, meaning that only 11% to 13% of the BHB concentration can be explained by the factors studied in our analyses, which might be due to the complex interplay of hormones, metabolism, and dietary factors. Furthermore, the large sample size increases statistical power, which can result in statistically significant associations that may not be clinically or biologically meaningful. To minimize this risk, we selected variables that have been suggested to be associated with BHB based on prior literature or that theoretically may be associated. Another limitation is that most participants in the PREVEND study are of Northern European origin, which limits the generalizability of these results to populations of different ethnic backgrounds. Furthermore, we did not validate these findings in a different cohort. Additionally, dietary patterns were not recorded in detail during this study. For future studies, it might be of interest to study the determinants of ketone body levels during a ketogenic diet trial.

In conclusion, we found statistically significant associations between various factors and BHB levels, including sex, hormonal status, glucose metabolism, kidney function, protein and sodium intake, and alcohol consumption, in a large population cohort. Notably, we found an interaction between sex and age for their association with BHB, with BHB levels increasing more strongly with age in men. In women, OC use and menopause state were associated with BHB concentrations, suggesting a potential influence of female sex hormones on ketogenesis. These factors could be used in future trials of ketogenic interventions to stratify patients. The findings of this comprehensive analysis emphasize the complex interplay of metabolic, hormonal, and lifestyle factors in regulating ketone body concentrations, particularly in the context of sex differences.

## Data Availability

Some or all datasets generated during and/or analyzed during the current study are not publicly available but are available from the corresponding author on reasonable request.

## Abbreviations

AcAc: acetoacetate
BHB: β-hydroxybutyrate
BMI: body mass index
eGFR: estimated glomerular filtration rate
FFAs: free fatty acids
FT4: free thyroxine
GH: growth hormone
HDL: high-density lipoprotein
IQR: interquartile range
NMR: nuclear magnetic resonance
NT-proBNP: N-terminal pro-B-type natriuretic peptide
OC: oral contraceptive
PREVEND: Prevention of Renal and Vascular End-stage Disease
T2D: type 2 diabetes
TSH: thyrotropin
